# Novel compound heterozygous stop‐gain mutations of *LRBA* in a Vietnamese patient with Common Variable Immune Deficiency

**DOI:** 10.1002/mgg3.1216

**Published:** 2020-03-10

**Authors:** Anh N. L. Phan, Thuy T. T. Pham, Nghia Huynh, Tuan M. Nguyen, Cuc T. T. Cao, Duong T. Nguyen, Duc T. Le, Chi‐Bao Bui

**Affiliations:** ^1^ Children’s Hospital 1 Ho Chi Minh City Vietnam; ^2^ Functional Genomic Unit DNA Medical Technology Ho Chi Minh City Vietnam; ^3^ Department of Hematology Ho Chi Minh City University of Medicine and Pharmacy Ho Chi Minh City Vietnam; ^4^ Biomedical Research Center School of Medicine, Vietnam National University HCMC Ho Chi Minh City Vietnam; ^5^ Molecular Genetics City Children’s Hospital Ho Chi Minh City Vietnam

**Keywords:** antibody deficiency, chronic diarrhea, common variable immunodeficiency, *LRBA*, stop‐gain mutation

## Abstract

**Background:**

Lipopolysaccharide‐responsive and beige‐like anchor (LRBA) deficiency is a rare autosomal recessive common variable immunodeficiency (CVID), affecting 1:25,000–1:50,000 people worldwide. Biallelic mutations in the gene *LRBA* have been implicated in affected individuals.

**Methods:**

We report a 16‐year‐old Vietnamese, male patient with recurrent CVID symptoms including chronic diarrhea, interstitial pneumonia, cutaneous granulomatous lesions, hepatosplenomegaly, and finger clubbing. Immunological analyses and whole exome sequencing (WES) were performed to investigate phenotypic and genotypic features.

**Results:**

Immunological analyses revealed hypogammaglobulinemia and low ratios of CD4+/CD8+ T cells. Two novel compound heterozygous stop‐gain mutation in *LRBA* were identified: c.1933C > T (p.R645X) and c.949C > T (p.R317X). Sanger sequencing confirmed the segregation of these variants from the intact parents. The abolished LRBA protein expression was shown by immunoblot analysis. Subsequent treatment potentially saves the child from the same immune thrombocytopenia which led to his brother's untimely death; likely caused by the same *LRBA* mutations.

**Conclusion:**

This first report of LRBA deficiency in Vietnam expands our knowledge of the diverse phenotypes and genotypes driving CVID. Finally, the utilization of WES shows great promise as an effective diagnostic for CVID in our setting.

## Graphical abstract

We describe the clinical, immunological, and genetic analyses of a Vietnamese patient presenting with typical and atypical symptoms of common variable immune deficiency. Whole exome sequencing revealed novel compound stop‐gain mutations of LRBA. Our findings also expand the already broad range of known CVID clinical phenotypes and contribute to the understanding of phenotype–genotype correlation in LRBA deficiency.

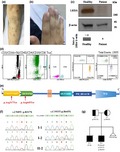

## 1 INTRODUCTION

Common variable immunodeficiency (CVID) is the most prevalent primary immunodeficiency disorder, presenting profound heterogeneity in phenotype and genotype (Gathmann et al., 2014). CVID is characterized by low levels of antibody leading to recurrent respiratory infections; chronic lung, autoimmune, gastrointestinal, and granulomatous diseases as well as lymphoma and solid tumors (Gathmann et al., 2014). 10%–20% of CVID cases are explained by mutations in one of a number of genes including lipopolysaccharide‐responsive and beige‐like anchor (*LRBA*; OMIM #614700; Eren Akarcan et al., 2018; Hou et al., 2017).

Located on locus 4q31.3, *LRBA* is a member of the highly conserved WDL‐BEACH‐WD (WBW) gene family. *LRBA* is widely distributed across many tissues and cell types but with particularly high expression in T and B cells (Wu et al., 2009). *LRBA* plays a role in coupling signal transduction and vesicle trafficking, leading to polarized secretion and/or membrane deposition of immune effector molecules (Cullinane, Schäffer, & Huizing, 2013; Wang, Howson, Haller, & Kerr, 2001). Unsurprisingly, *LRBA* is instrumental in immune regulation, host defense, cell proliferation, and cell death (Gámez‐Díaz et al., 2016; Habibi et al., 2019; Lopez‐Herrera et al., 2012). LRBA deficiency is a rare autosomal recessive disorder caused by biallelic mutations in the *LRBA* gene. There is no clear genotype–phenotype correlation in LRBA‐deficient patients who present with a spectrum of diverse mutations, immunologic characteristics, and clinical symptoms (Gámez‐Díaz et al., 2016; Kostel Bal et al., 2017; Lopez‐Herrera et al., 2012)_._

Detection of LRBA deficiency is commonly by flow cytometry or western blotting with subsequent validation by genetic testing (Azizi et al., 2018; Gamez‐Diaz et al., 2018; Lanio, Sarmiento, Gallego, & Carbone, 2009). More recently, next‐generation sequencing technology has enabled the fast elucidation of CVID genetic etiologies of either known or unknown phenotypes (Maffucci et al., 2016). Herein, we report a case of a 16‐year‐old boy with CVID resulting from novel compound heterozygous mutations in *LRBA* that we diagnosed using a WES‐based approach. His presentation of both classical and uncommon symptoms expands the broad range of CVID clinical phenotypes and adds to the understanding of phenotype–genotype correlation in LRBA deficiency.

## 2 MATERIALS AND METHODS

### 2.1 Patient recruitment and consent

Clinical data were obtained from the patient's medical records. Written informed consent for this case report and publication was provided by the patient's parents under the Ethics Review Board of Children's Hospital 1 Ho Chi Minh City and Ho Chi Minh City University of Medicine and Pharmacy, Vietnam.

### 2.2 Immunological analysis

The serum immunoglobulin level was measured by nephelometric technique. The lymphocyte subsets from the peripheral blood samples were analyzed by fluorescence‐activated cell sorting using the BD Multitest™ reagents (BD Multitest IMK kit and BD Multitest™ 6‐color TBNK).

### 2.3 Genetic analysis

Genomic DNA was extracted from peripheral blood using QIAamp DNA Blood Mini Kit (#51104, QIAGEN, Netherlands) and then whole exome library preparation and sequencing was performed by Macrogen (South Korea), using Agilent SureSelect Human All Exon V5 (Agilent Technologies) on a NovaSeq 6000 Sequencing System (Illumina). We applied an in‐house bioinformatics pipeline for WES analysis.

### 2.4 Immunoblot

Protein lysates derived from peripheral blood mononuclear cell (PBMC) of the patient and control, were resolved on Tris‐acetate gel 3%–8% (NuPAGE, Thermofisher scientific), transferred to nitrocellulose filters and then blotted with polyclonal rabbit anti‐LRBA antibodies (Abiocode) and monoclonal mouse anti–β actin (Sigma).

## 3 RESULTS

### 3.1 Clinical presentation

**Figure 1:**
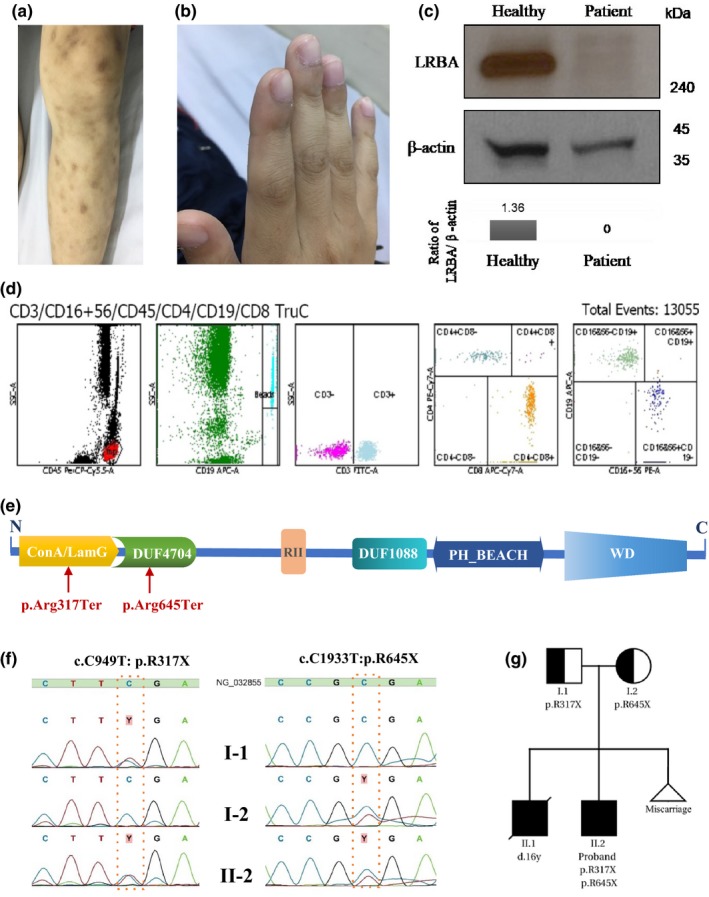
Clinical presentation, immunological, and genetic analyses of the patient. (a) Cutaneous granulomatous lesions and (b) Finger clubbing in the patient. (c) Detection by immunoblot of LRBA from the patients’ and healthy person's PBMC. Normalization was performed with β‐actin. (d) Lymphocyte immunophenotyping of total T lymphocytes, CD3^+^ T cells, CD4^+^ T cells, CD8^+^ T cells, B cells, and natural killer cells. (d) Lymphocyte immunophenotyping of total T lymphocytes, CD3^+^ T cells, CD4^+^ T cells, CD8^+^ T cells, B cells, and natural killer cells. (e) Schematic representation of *LRBA* protein domains and identified variants. BEACH, Beige/BEACH domain; DUF, the domain of unknown function; Lam G, laminin G domain; PH, PH domain associated with beige/BEACH; WD, WD‐40 repeat; RII, protein kinase A regulatory subunit binding motifs (f) Sanger sequencing of *LRBA* in the proband and both parents. (g) Pedigree of the patient and parent's: the proband's brother died at 16 years old with chronic immune thrombocytopenic purpura, the mother also had a miscarriage after the proband. Generations and subjects are depicted in Roman numerals (I–II) and Arabic numerals. Circles, female subjects; squares, Male subjects; solid symbols, patients

In 2018, a male patient was first admitted to the inpatient clinic at age 14 with chronic diarrhea and failure to thrive with weight and height under −2 *SD* score. He had experienced diarrhea, fatigue, and weight loss for 3 years preadmission. Past medical history included chickenpox and measles. The patient got MMR vaccination in 2014 with no complications. There had also been no lymphadenitis after BCG vaccination. The patient was born as the second child of nonconsanguineous healthy Vietnamese parents whose brother had died of chronic immune thrombocytopenic purpura (ITP) at 16 years old. CVID was diagnosed by the typical clinical manifestations of diarrhea, failure to thrive, and interstitial pneumonia. Unusually, since he was 7 years old, he also had multiple persistent cutaneous granulomatous lesions, erythematous papules, and small plaques that enlarged gradually on his limbs (Figure 1a), finger clubbing (Figure 1b), and hepatosplenomegaly without lymphadenopathy. In addition, stool examinations for lipid, blood, parasites, and viruses, as well as tuberculosis profile and HIV test were negative. No infectious etiologies were identified. The abdominal and thoracic CT scan revealed interstitial pneumonia, colitis, and inflammation of the mesenteric ganglia. Colonoscopy and esophagogastroduodenoscopy showed no abnormalities; however, biopsy results revealed chronic gastritis and atypical colitis. Peripheral blood leukocyte count was in normal range and erythrocyte sedimentation rate did not increase.

### 3.2 Immunological analyses and clinical treatment

Standard immunological workup showed C3, C4, ANA, anti‐dsDNA, and ANA‐8 parameters profile to be within normal range. Immunoglobulin quantification results showed low IgG, IgA, IgM, and IgE levels (Table 1). Furthermore, the hepatitis B surface antibody test remained negative after three doses of hepatitis B vaccine. Lymphocyte immune‐phenotyping revealed normal counts for total T cells, CD3^+^, CD4^+^, and CD8^+^ T cells; B cells; and natural killer cells (Table 1). The patient also had reduced CD4/CD8 T cell ratio (Table 1; Figure 1d).

**Table 1:** Immunological profile and *LRBA* mutations of patient

Immunological work‐up		
Parameters	Measurement at age 14	Reference range
Lymphocyte (×10^3^/µL)	2.42	1.2–5.2
IgA (mg/dl)	1.24 ↓	56–203
IgG (mg/dl)	225 ↓	660–1,220
IgM (mg/dl)	22 ↓	57–162
IgE (UI/mL)	<1.5 ↓	7–280
CD3 + T (cell/µL)	1,407	1,000–2,200
CD4 + T (cell/µL)	554	530–1,300
CD8+ T (cell/µL)	810	330–920
CD19 + B (cell/µL)	232	110–570
CD56 + NK (cell/µL)	192	70–480
CD4+/CD8+	0.68 ↓	1.1–1.4

ACMG‐AMP Classification of identified causative mutations in LRBA		
ACMG‐AMP Criteria	*LRBA*:NM_001199282: exon8:c.C949T:p.R317X	*LRBA*:NM_001199282: exon15:c.C1933T:p.R645X
PVS1	Stop‐gain mutation (LamG_3 domain)	Stop‐gain mutation (DUF4704 domain)
PM2	GnomAD (absent)	GnomAD (0.00000817)
PM3	For a recessive disorder, 2 variants detected in trans by Sanger sequencing	
PP3	GERP++ (0.782)	GERP++ (0.586)
	DANN (0.886)	DANN (0.863)
	CADD (0.987)	CADD (0.985)
	MutationTaster (0.81, Disease causing automatic)	MutationTaster (0.81, Disease causing automatic)
	FATHMM‐MKL (0.618, Damaging)	FATHMM‐MKL (0.548, Damaging)
	LRT (0.441, Deleterious)	LRT (0.629, Deleterious)
	EIGEN (0.846, Pathogenic)	EIGEN (0.6959, Pathogenic)
PP4	Patient's phenotype and family history (the brother died at age 16) is highly specific for CVID with a single genetic etiology	

Note

DUF4704, the domain of unknown function; LamG_3, Laminin G‐like domain. The numbers in bracket indicate the in silico prediction converted rank‐score and prediction classifier (if available), respectively. ↑ and ↓ indicate a value above and below the reference ranges, respectively.

Prophylactic treatment with antibiotics (trimethoprim and dapsone) and an antifungal (fluconazole) was initiated for infection control but antibiotics were ceased due to an allergic reaction. A subsequent prophylactic regimen of intravenous immunoglobulin (IVIG) therapy and fluconazole was carried out for 4 weeks. At 1‐year follow‐up, no severe infections or malignancies were reported. Recently, immune thrombocytopenia (ITP) was diagnosed and treated promptly with IVIG (500 mg/kg). Platelet count rose following this treatment remaining stable (34 × 103/µl) at 2‐week follow‐up.

### 3.3 Genetic and immunoblot analyses

We implemented WES to investigate the underlying genetic cause of CVID in the patient, following ACMG‐AMP guidelines for variant classification (Richards et al., 2015). A number of candidate variants in primary immunodeficiency genes were detected including two novel heterozygous variants in *LRBA*: c.1933C > T, (p.R645X) and c.949C > T, (p.R317X; Table S1). While these 2 *LRBA* mutations could explain our patient phenotype, the remaining variants did not match his clinical presentation (Table S1). c.C1933T, (p.R645X) is located on exon 15, within the domain of unknown function DUF4704, while c.C949T, (p.R317X) affects the Laminin G‐like domains (LamG) of LRBA (Figure 1e). Sanger sequencing confirmed the compound heterozygous mutations in the patient which was likely inherited from both of his carrier parents (Figure 1f,g). While c.949C > T is absent in GnomAD database, c.1933C > T has an MAF of 0.00000817 (smaller than 0.0001 threshold for recessive genes; Table 1). Both variants have pathogenic potential as per computational analysis from DANN, GERP++, LRT, MutationTaster and FATHMM‐MKL coding, CADD, and EIGEN score (Table 1). Additionally, both of our variants are absent in Vietnamese Genetic Variation Database (includes 305 genomes and exomes of healthy Vietnamese (Vinh et al., 2019) and in current version of Genome Asia 100K browser (retrieved February 17 2020, from https://browser.genomeasia100k.org). In brief, the two variants were defined as pathogenic as they both meet ACMG classification criteria PVS1, PM2, PM3, PP3, and PP4 (Li & Wang, 2017). Moreover, copy number variant analysis based on WES data revealed no relevant variants. The western blot analysis revealed the absence of LRBA protein expression in the patient (Figure 1c).

## 4 DISCUSSION

The etiology of CVID in a 16‐year‐old Vietnamese patient was investigated by clinical, immunological, and genetic analyses. Using our in‐house bioinformatics pipeline for WES analysis, we report novel biallelic stop‐gain mutations in the gene *LRBA* which is in fitting with the clinical presentation of the patient. Our novel *LRBA* mutation discovery and clinical history adds to the knowledge of diverse genotypes and phenotypes driving CVID.

WES analysis identified variant p.R645X within the domain of unknown function (DUF4704), which is found on neurobeachin proteins in eukaryotes. A neurobeachin/LRBA homolog in *Caenorhabditis elegans* (SEL‐2), is known to take part in endosomal traffic and delivery of cell surface proteins to the lysosome. The second mutation p.R317X affects the Laminin G‐like domain (LamG) of LRBA. These domains are often Ca^++^ mediated receptors with binding sites for steroids, beta‐1 integrins, heparin, sulfatides, fibulin‐1, and alphadystroglycans. Proteins containing LamG domains, such as LRBA, are involved in cellular process such as signal transduction, adhesion, migration, and differentiation. The western blot analysis showed the absent of LRBA protein in our patient. This is in agreement with several previous studies demonstrating the abolished expression of LRBA protein in patients with deleterious mutations (Alkhairy et al., 2016; Azizi et al., 2018; Seidel et al., 2018). Our two truncating mutations, located close to the N‐terminal of protein, likely remove critical domains and affect protein kinase regulatory subunit (RII) binding motifs, resulting in loss‐of‐function in *LRBA*. The two variants are in close proximity to mutations found in LRBA‐deficient patients in the study of Charbonnier et al. (2015) and Lopez‐Herrera et al. (2012), in which the patients share similar clinical features, including immune dysregulation and Treg deficiency; polyendocrinopathy and enteropathy. Enteropathy was more frequently observed among patients with nonsense or insertion/deletion mutations clustering in or before the Beige/BEACH domain (BEACH) (Figure 1e) similarly to our patient (Habibi et al., 2019). This implies a possible association between certain *LRBA* mutations and severe manifestations like enteropathy (Habibi et al., 2019).

LRBA‐deficient patients have been reported with a spectrum of mutations and with highly variable clinical and immunologic characteristics. Classical clinical manifestations of LRBA deficiency was evident in our patient including chronic diarrhea, failure to thrive, and interstitial pneumonia. Uncommon symptoms were also observed including multiple persistent cutaneous granulomatous lesions on his limbs, finger clubbing, and hepatosplenomegaly although the latter has previously been reported in a few patients (Alkhairy et al., 2016; Eren Akarcan et al., 2018). The main clinical complications of LRBA deficiency have been described as immune dysregulation (95%), organomegaly (86%), recurrent infections (71%), and hypogammaglobulinemia (57%) (Gámez‐Díaz et al., 2016) all of which were evident in our patient. Additionally, many LRBA‐deficient patients (Lanio et al., 2009) suffer from at least one autoimmune disorder with autoimmune hemolytic anemia and ITP the most common, followed by autoimmune thyroid disease, autoimmune enteropathy, type 1 diabetes mellitus, and juvenile idiopathic arthritis (Charbonnier et al., 2015; Kostel Bal et al., 2017). Our patient presented with multiple persistent cutaneous granulomatous lesions on his limbs and laterally, development of ITP. The patient's brother died at 16 years old due to ITP. The similar clinical history of the siblings, in combination with the *LRBA* mutation family segregation, suggests the existence of these mutations in the deceased brother who had not undergone genetic analysis.

The presentation of normal B cell count and negative hepatitis B surface antibody following vaccination in our patient is also in line with 42% of patients reported in the same study (Gámez‐Díaz et al., 2016). Our patient presented with hypogammaglobulinemia and decreased CD4^+^/CD8^+^ T cell ratio. While another study by Gámez‐Díaz et al., reported normal levels of CD4 and CD8 T cells in up to 81% of patients, 25%–30% often had increased numbers of CD8^+^ T cells and a declined CD4^+^/CD8^+^ ratio (<1; Gámez‐Díaz et al., 2016). Many in vitro studies of cells from LRBA‐deficient patients also showed decreased IgG antibody production, defective T‐cell activation and proliferation, increased apoptosis, and decreased autophagy in B lymphocytes of affected individuals (Alroqi et al., 2018; Charbonnier et al., 2015; Gámez‐Díaz et al., 2016; Hou et al., 2017; Lopez‐Herrera et al., 2012). LRBA‐deficient individuals are currently treated with a wide range of therapies. Of the 22 patients in one study, two were treated successfully with hematopoietic stem cell transplantation, seven with immunoglobulin replacement, and 15 with immunosuppressive treatment (Gámez‐Díaz et al., 2016). As of today, there is no controlled study of whether HSCT or abatacept is the preferable treatment option in LRBA deficiency (Kostel‐Bal et al., 2017). In a latest multicenter study of 76 LRBA‐deficient patients, overall survival in 24 patients underwent HSCT was 70.8% (median follow‐up 20 months). Of 17 HSCT survivors, seven were in complete and five in good partial remission without treatment. Only five of 43 nontransplant patients (11.6%) are without immunosuppression with abatacept or rapamycin (Tesch et al., 2019). In accordance, our patient was treated successfully with IVIG and remained asymptomatic after 1‐year follow‐up but begun to present ITP recently. Within our condition, we have not got abatacept or rapamycin, HSCT is a bridging strategy for him. The treatment potentially saves the child from the same ITP which led to his brother's untimely death; likely caused by the same *LRBA* mutation. Further work is clearly needed to investigate and personalize therapies based on the genetic and phenotypic characterization of CVID.

In conclusion, by applying WES analysis we identified novel *LRBA* mutations as the genetic cause of CVID in a 16‐year‐old boy. This compound heterozygous mutation in *LRBA* is highly associated with common features of CVID. Furthermore, we also report unique clinical manifestations which adds to the growing body of knowledge describing the phenotypically and genetically diverse set of diseases driven by LRBA deficiency. The pathogenicity of these *LRBA* variants is validated firstly by immunoblotting showing the absent expression of LRBA protein. Finally, these findings suggest that genetic analysis facilitates early diagnosis and optimal treatment for severe and life‐threatening CVID.

## AUTHOR CONTRIBUTIONS

Anh N L Phan was the principal clinician in charge of the patient's care and wrote part of the manuscript. Chi‐Bao Bui and Thuy T T Pham implemented the analysis of whole exome sequencing and Sanger sequencing, and wrote part of the manuscript. Nghia Huynh performed the immunological analysis. Tuan M Nguyen, Cuc T T Cao, and Duong T Nguyen were involved in management of the patient. All authors reviewed the manuscript and contributed to the final manuscript. The authors declare that no commercial or financial relationships that could be construed as a conflict of interest.

## Supporting information

Supplementary Material
