# Supplementary material for: Novel compound heterozygous stop‐gain mutations of LRBA in a Vietnamese patient with Common Variable Immune Deficiency
Source: Mol Genet Genomic Med. 2020 Mar 10;8(5):e1216. doi: 10.1002/mgg3.1216 (PMC7216813; doi:10.1002/mgg3.1216)
Supplement: Supplementary file 1 — Supplementary Material [file MGG3-8-e1216-s001.docx]

**SUPPORTING MATERIALS AND METHODS**

**Whole exome sequencing (WES)**

Genomic DNA was extracted from peripheral blood using QIAamp DNA Blood Mini Kit (#51104, QIAGEN, Netherland). Qualified DNA was subsequently examined for quality using DropSense96, Qubit 2.0, TapeStation. The ideal range for these parameters is: Chemical contaminants A260/A230: 1.50 – 2.50; protein contaminants A260/A280: 1.60 - 2.20; Concentration > 1 ng/µL; DNA smear (50% > 1000 bp). Whole exome library preparation and sequencing was performed by Macrogen (South Korea), using Agilent SureSelect Human All Exon V5 (Agilent Technologies, Santa Clara, California, USA) on a NovaSeq 6000 Sequencing System (Illumina, Seoul, South Korea).

Paired FASTQ reads were processed by FASTQC to obtain useful diagnostics such as Phred-score distribution along the reads, GC content distribution, read-length distribution, sequence duplication level. Trimmomatic was subsequently used for removing contaminated sequencing adapters; removing leading and trailing low quality or N bases below quality 3; scanning the read with a 4-base wide sliding window, cutting when the average quality per base drops below 15; and dropping reads below a length of 36. Pre-processed read pairs were then mapped to hg19 reference genome (from UCSC) by BWA-mem. Additional processing included: MarkDuplicates by PICARD, BaseQualityScoreRecalibration by GATK. VCF files were generated with GATK HaplotypeCaller; filtrated by GATK VariantFiltration, SNP (QD < 2.0, FS > 60.0, MQ < 40.0, MQRankSum < -12.5, ReadPosRankSum < -8.0) and INDEL (QD < 2.0, FS > 200.0, ReadPosRankSum < -20.0), respectively. Finally, ANNOVAR was used to intersect variant annotations from UCSC RefSeq, dbSNP 150, gnomAD, ESP6500, ExAC, 1000G, dbNSFPv3.5. We applied an in-house bioinformatics pipeline for WES analysis which integrates collaboration between geneticists and physicians. We used a gene panel for primary immunodeficiency including an extensive list of disease-causing genes based on current clinical genetic database: Clinical Genomic Database, MALACARD, OMIM, GHR. Rare variants with MAF<0.01 in several population databases were selected for further analysis. dbNSFP was utilized to identify top candidate variants by compiling prediction scores from 29 algorithms.

**Sanger sequencing validation**

The family segregation of all detected variants was validated by Sanger sequencing. PCR was performed using forward and reverse primers for variants on gene *LRBA*: c.C949T (F: 5′- GCTTCTGCAGTGGTATATGG- 3′; R: 5′-ACCCTATAGTGGAGCTCTTG-3′), c.C1933T (F:5′-CACATGGAAAAGCTGTTGGG-3′; R:5′- GCAACGTGAACTCACCTTCA -3′). Following amplification, PCR products were treated with Exo-SAP for use in Big Dye Terminator v3.1 Cycle Sequencing. Sequencing data was subsequently analyzed on the 3500 Genetic Analyzer (ThermoFisher Scientific, Life Technologies) and assessed on CLC Main Workbench 8.1. software.

**SUPPORTING FIGURE LEGENDS**

**Table S1.** List of candidate variants detected using WES.

| **Chr** | **Start** | **Ref** | **Alt** | **Func. refGene** | **AAChange.refGene; Interpro_domain** | **ExAC** | **Zygosity** | Gene | **Clinical implication** |
| --- | --- | --- | --- | --- | --- | --- | --- | --- | --- |
| 4 | 151818962 | G | A | exonic | NM_001199282:exon15:c.C1933T:p.R645X, Domain of unknown function DUF4704 | 8.62E-06 | Het | LRBA | Immunodeficiency, common variable, 8, with autoimmunity |
| 4 | 151836825 | G | A | exonic | NM_001199282:exon8:c.C949T:p.R317X, Concanavalin A-like lectin/glucanase domain | . | Het | LRBA | Immunodeficiency, common variable, 8, with autoimmunity |
| 3 | 187003786 | C | T | exonic | NM_001031849:exon2:c.G64A:p.V22M, CUB domain | 0.0012 | Het | MASP1 | Mannan-binding lectin serine protease (MASP) deficiency |
| 5 | 94834113 | C | T | exonic | NM_014639:exon33:c.G3524A:p.S1175N, Tetratricopeptide-like helical domain | 3.30E-05 | Het | TTC37 | Trichohepatoenteric syndrome 1 |
| 11 | 60229885 | C | T | exonic | NM_021950:exon2:c.C38T:p.P13L | 0.0001 | Het | MS4A1 | Immunodeficiency, common variable, 5 |
| 16 | 14687223 | T | C | exonic | NM_001242992:exon12:c.A715G:p.I239V; R3H domain;Ribonuclease H-like domain | 5.02E-05 | Het | PARN | Dyskeratosis congenita, autosomal recessive 6; Pulmonary fibrosis and/or bone marrow failure,telomere-related 4 |
| 17 | 78182049 | G | A | exonic | NM_024110:exon21:c.G2920A:p.G974S; Guanylate kinase-like domain | 9.58E-05 | Het | CARD14 | CARD14 mediated psoriasis;Psoriasis 2; Pityriasis rubra pilaris |
| 22 | 40814706 | T | C | exonic | NM_001282660:exon9:c.A1736G:p.N579S | 0.0002 | Het | MKL1 | Megakaryoblastic leukemia, acute |
| 8 | 61653981 | G | A | UTR5 | . | 4.18E-05 | Het | CHD7 | Charge syndrome;CHARGE syndrome ; Immunodeficiency |
| 19 | 10461491 | G | A | UTR3 | . | 0.0008 | Het | TYK2 | Immunodeficiency 35 611521;Hyper IgE syndrome (HIES) |
| 17 | 16842773 | TCA | - | UTR3 | . | . | Hom | TNFRSF13B | Immunodeficiency, common variable, 2; Immunoglobulin A deficiency 2 |
| 8 | 42188927 | A | C | UTR3 | . | . | Het | IKBKB | Immunodeficiency 15 |
| 1 | 948870 | C | G | UTR5 | . | . | Hom | ISG15 | Immunodeficiency 38 |
| 1 | 22987958 | A | G | UTR3 | . | . | Hom | C1QB | Immunodeficiency due to an early component of complement deficiency |
| 1 | 57395020 | A | G | UTR3 | . | . | Het | C8B | Complement component 8 deficiency |

* The primary immunodeficiency diseases panel of 303 genes following International Union of Immunological Societies (IUIS, 2017) is used to screen for candidate variants.
